# Radiomics as a measure superior to common similarity metrics for tumor segmentation performance evaluation

**DOI:** 10.1002/acm2.14442

**Published:** 2024-06-23

**Authors:** Rukhsora Akramova, Yoichi Watanabe

**Affiliations:** ^1^ Department of Radiation Oncology University of Minnesota Minneapolis Minnesota USA

**Keywords:** Dice similarity coefficient (DSC), Hausdorff distance (HD), intraclass correlation coefficient (ICC), radiomics features, segmentation evaluation, surface Dice similarity coefficient (sDSC)

## Abstract

**Purpose:**

To propose radiomics features as a superior measure for evaluating the segmentation ability of physicians and auto‐segmentation tools and to compare its performance with the most commonly used metrics: Dice similarity coefficient (DSC), surface Dice similarity coefficient (sDSC), and Hausdorff distance (HD).

**Materials/methods:**

The data of 10 lung cancer patients’ CT images with nine tumor segmentations per tumor were downloaded from the RIDER (Reference Database to Evaluate Response) database. Radiomics features of 90 segmented tumors were extracted using the PyRadiomics program. The intraclass correlation coefficient (ICC) of radiomics features were used to evaluate the segmentation similarity and compare their performance with DSC, sDSC, and HD. We calculated one ICC per radiomics feature and per tumor for nine segmentations and 36 ICCs per radiomics feature for 36 pairs of nine segmentations. Meanwhile, there were 360 DSC, sDSC, and HD values calculated for 36 pairs for 10 tumors.

**Results:**

The ICC of radiomics features exhibited greater sensitivity to segmentation changes than DSC and sDSC. The ICCs of the wavelet‐LLL first order Maximum, wavelet‐LLL glcm MCC, wavelet‐LLL glcm Cluster Shade features ranged from 0.130 to 0.997, 0.033 to 0.978, and 0.160 to 0.998, respectively. On the other hand, all DSC and sDSC were larger than 0.778 and 0.700, respectively, while HD varied from 0 to 1.9 mm. The results indicated that the radiomics features could capture subtle variations in tumor segmentation characteristics, which could not be easily detected by DSC and sDSC.

**Conclusions:**

This study demonstrates the superiority of radiomics features with ICC as a measure for evaluating a physician's tumor segmentation ability and the performance of auto‐segmentation tools. Radiomics features offer a more sensitive and comprehensive evaluation, providing valuable insights into tumor characteristics. Therefore, the new metrics can be used to evaluate new auto‐segmentation methods and enhance trainees' segmentation skills in medical training and education.

## 1 INTRODUCTION

Many new auto‐segmentation methods using artificial intelligence technology are being developed.^1^, ^2^, ^3^ To evaluate the performance of new tools, the need for a more sensitive and informative evaluation tool for tumor segmentation in medical imaging has become increasingly apparent.^4^

There are standard methods to check the geometrical correctness and accuracy of segmentation.^5^ The Dice similarity coefficient (DSC), the surface Dice similarity coefficient (sDSC), and the Hausdorff distance (HD) are frequently employed to assess the similarity by quantifying the overlap between two segmentations.^5^, ^6^, ^7^ While the above geometric measures are widely used to evaluate the similarity between segmented and reference volumes, it has several limitations to consider when interpreting their results.^5^, ^6^ DSC treats all disagreements between the segmented volume and the reference volume equally, regardless of whether errors are systematic (consistent across multiple cases) or random (vary between cases). As a result, it cannot differentiate between these error types, potentially leading to misleading interpretations of the segmentation quality. sDSC needs a tolerance threshold which strongly affects the calculated sDSC value. HD is sensitive to local differences between two contours but fails to account for the difference in the entire contours.^5^

The abovementioned limitations highlight the need for a comprehensive evaluation beyond the DSC, sDSC, and HD when assessing segmentation quality. We have identified radiomics features as a more comprehensive approach that provides valuable information about tumor size, shape, intensity, and texture characteristics. Considering the comprehensiveness of radiomics in evaluating image characteristics,^8^ we hypothesize that radiomics features can offer a convenient means to compare and assess segmentations. Using the public data library Reference Database to Evaluate Response (RIDER),^9^ we demonstrate the superiority of radiomics features compared with the common similarity metrics to evaluate the difference in segmentation.

## 2 METHODS AND MATERIALS

### 2.1 Data

This study used publicly available data kept in the RIDER data library.^10^ Only lung data were downloaded for the analysis. The data consisted of CT dataset with nine segmentations made by three institutions using auto‐segmentation tools. Each institution did tumor segmentation using institution‐specific software by setting three different segmentation parameters. The CT dataset was prepared for 10 patients.^9^ Consequently, our data set contained 10 CT datasets and 90 segmentation data of lung tumors drawn on the CT images.^11^

### 2.2 Radiomics

**FIGURE 1:**
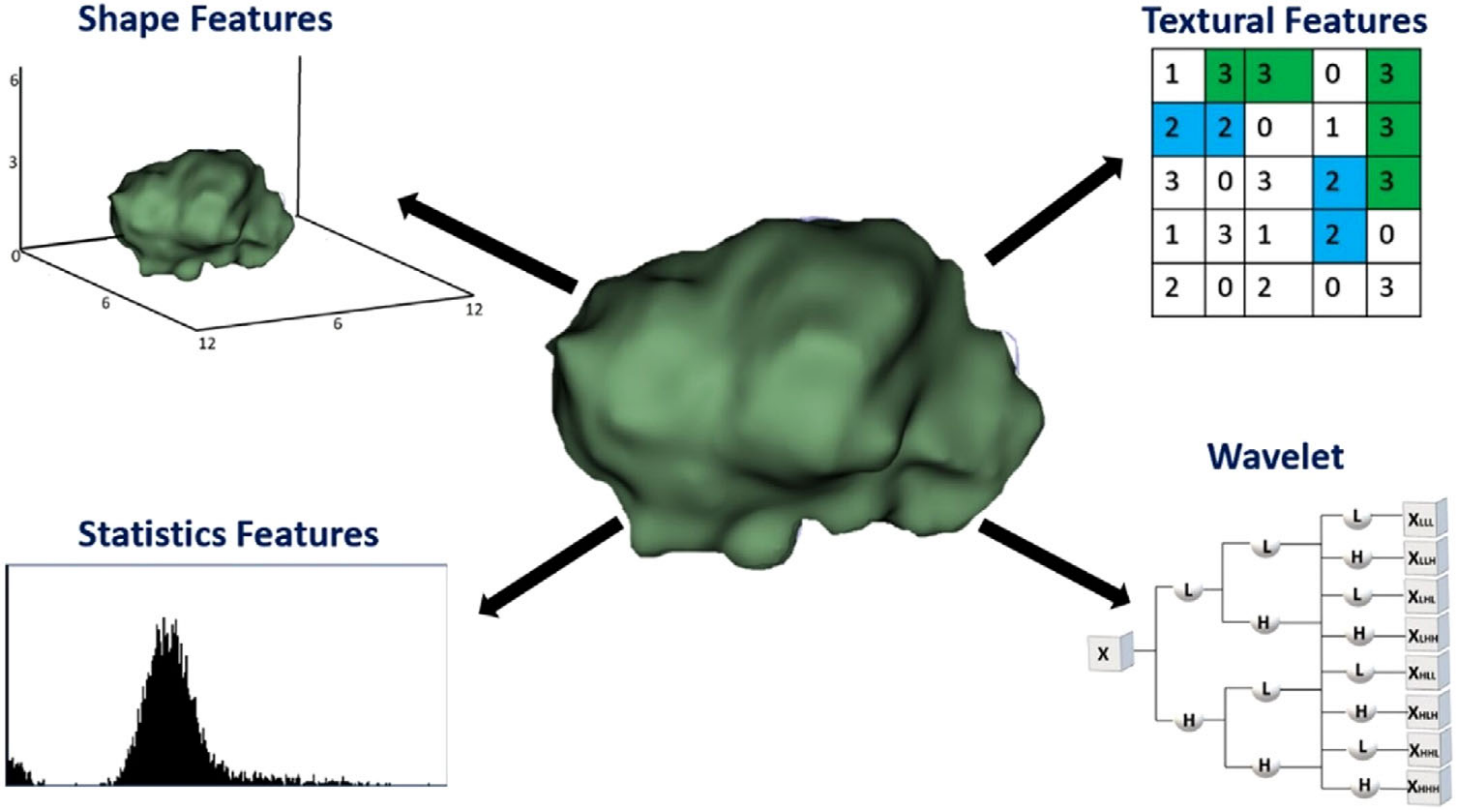
Radiomic features represent a tumor's intensity (statistics), shape, and texture, providing a comprehensive overview of its properties. CT images were resampled with 2 mm voxel size and filtered by LoG 1,1,1 mm and wavelets.

Radiomics utilizes mathematical formulas to characterize the shape, intensity, and texture with additional image filters,^12^ as illustrated in Figure 1. See Section A in the supplemental materials for the meanings of radiomics features. This study employed 3D Slicer^13^ for radiomics feature extraction and DSC, sDSC, and HD calculations of the RIDER lung image data. The Radiomics module in 3D Slicer, SlicerRadiomics, is a specialized component that facilitates the extraction and analysis of radiomics features from medical images. It is a 3D Slicer implementation of PyRadiomics.^14^ The module allows users to load medical image data (DICOM or other formats) and create or load segmentations representing regions of interest (ROIs) within the images. We calculated various radiomics features, including shape‐based, intensity‐based (or the first order), and texture‐based metrics. CT images were resampled with 2 mm voxel size and filtered by Gaussian‐of‐Laplacian (LoG) 1,1,1 mm and wavelets. The Bin width was set as 25. Nine hundred forty‐four radiomics features were obtained for each of the 90 image data sets (nine different segmentations for 10 patients).

### 2.3 Calculations of similarity metrics

**FIGURE 2:**
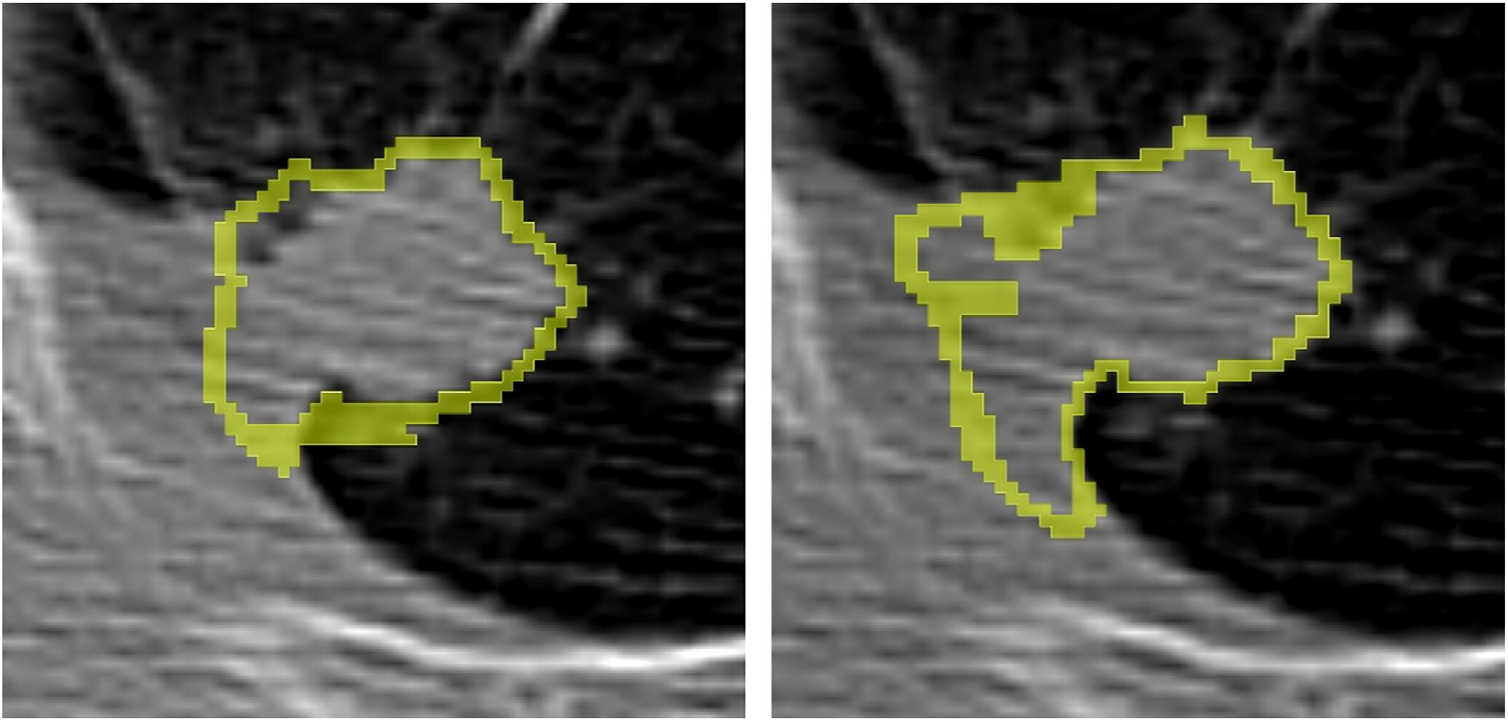
Two contours on the same CT image set of one patient are different.

Figure 2 shows two segmentations drawn for the same CT image data. We can easily recognize slight but non‐negligible differences in the tumor's shape. To quantify the difference between two segmentations drawn on the same image, we calculated DSC, sDSC, and HD for a pair of nine segmentations using the 3D Slicer, resulting in 36 values per patient. The calculations were repeated for all 10 patients. Hence, there were 36 × 10 values of DSC, sDSC, and HD.

For the radiomics‐based comparison, we used the intraclass correlation coefficient (ICC).^15^, ^16^ First, we obtained one ICC value, denoted as ICC*, per radiomics feature for 10 tumors and nine segmentations. Next, we calculated the ICC for 36 pairs of nine segmentations, resulting in 36 ICC values per radiomics feature. ICCs were calculated using a two‐way mixed model for absolute agreement.^16^

### 2.4 Statistical analysis

All statistical analyses were done using standard Python library routines and the ICC package available at GitHub.^17^

## 3 RESULTS

**FIGURE 3:**
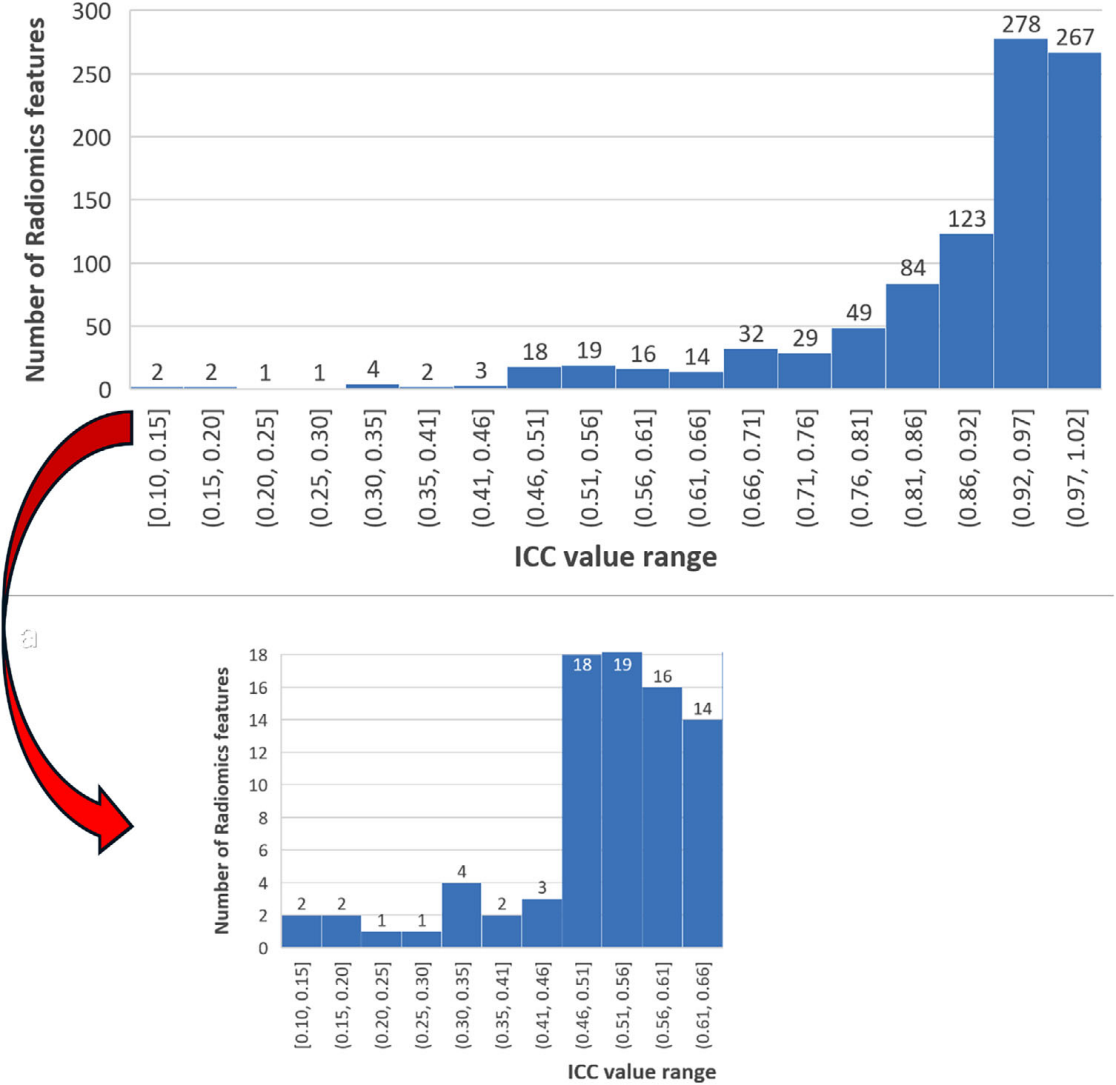
Histogram represents the distribution of 944 radiomics features. Part (a) illustrates the number of features across different value ranges. Part (b) presents a detailed view of the histogram, focusing specifically on the segment with the lowest ICC values, ranging from 0.1 to 0.51, featuring a count of only 33 features. ICC, intraclass correlation coefficient.

To compare nine segmentation methods applied to the CT data of 10 patients, we selected 10 radiomics features using ICC* values. Histograms in Figure 3 show the ICC* distribution of 944 radiomics features. Figure 3, Part (a) illustrates the number of features across different value ranges. Part (b) presents a detailed view of the histogram, focusing specifically on the segment with the lowest ICC* values, ranging from 0.1 to 0.51, which covered 33 radiomics features. The low ICC* indicates low reproducibility of feature values for different segmentations. Hence, we selected 10 radiomics features from those 33 features to enhance the detectability of differences among nine segmentation methods. Table 1 lists the 10 radiomics features selected for the analysis. The table included the ICC* values with the lower and upper 95% bound.

**TABLE 1:** ICC, lower and upper bound of 10 selected radiomics features with 95% confidence interval.

Image type	Feature class	Feature name	ICC*	ICC average	ICC min	ICC lower bound	ICC upper bound
wavelet‐LLL	glcm	ClusterShade	0.444	0.520	0.102	0.258	0.704
wavelet‐LLL	ngtdm	Complexity	0.339	0.351	‐0.133	0.172	0.612
wavelet‐LLL	glcm	MCC	0.344	0.391	‐0.214	0.175	0.616
original	glszm	GrayLevel Variance	0.326	0.365	‐0.159	0.163	0.600
wavelet‐HLL	gldm	Small DependenceHigh GrayLevelEmphasis	0.269	0.303	‐0.270	0.121	0.541
original	glcm	Cluster Prominence	0.236	0.301	‐0.219	0.100	0.501
wavelet‐LLL	first order	Maximum	0.371	0.506	0.157	0.192	0.647
wavelet‐HLL	glcm	MCC	0.393	0.398	‐0.243	0.215	0.663
wavelet‐LHH	first order	Median	0.420	0.461	‐0.058	0.235	0.687
wavelet‐HLL	glcm	Cluster Prominence	0.433	0.468	0.048	0.249	0.696

*Note*: ICC* is the ICC value of all nine segmentations. ICC average and ICC min are the average and the smallest of the ICCs for 36 pairs of 9 segmentations, respectively.

Abbreviation: ICC, intraclass correlation coefficient.

We also calculated 36 pair‐wise ICC values for 36 combinations of nine segmentations for the selected 10 radiomics features. The average and minimum of 36 ICC values are presented in Table 1. In addition, one DSC, sDSC, and HD values were calculated for each pair of segmentation per tumor; hence, there were 36 × 10 DSC, sDSC, and HD values for 10 tumors. For comparison with the radiomics method, we took an average of 10 tumors to have 36 DSC, sDSC, and HD values. Figure 4 shows three scatter plots with ICC in the abscissa and DSC, sDSC, and HD in the ordinate for 36 pairs of segmentations. The ICC of radiomics features exhibited greater sensitivity to segmentation changes than DSC and sDSC as the ICC values were wildly spread out, that is, from 0.0 to 1.0. In contrast, all DSC values were in a narrow range of 0.75 to 1.0 and all sDSC values were greater than 0.7. According to the standard evaluation system, all the segmentations are in good or very good agreement with DSC or sDSC > 0.7.^18^ On the other hand, ICC indicated that some segmentations were very different from others.^19^ For example, the ICCs of the wavelet‐LLL first order Maximum, wavelet‐LLL glcm MCC, wavelet‐LLL glcm ClusterShade features ranged from 0.130 to 0.997, 0.033 to 0.978, and 0.160 to 0.998, respectively. It is noted that HD ranged from 0 to 1.9 mm, as seen in Figure 4 c, indicating its high sensitivity to differences in segmentations.

FIGURE 4ICC of 10 radiomics features versus (a) DSC, (b) sDSC, and (c) HD. There are 36 ICC values per feature and 36 DSC, sDSC, and HD independent of the radiomics features. DSC, Dice similarity coefficient; HD, Hausdorff distance; ICC, intraclass correlation coefficient; sDSC, surface Dice similarity coefficient.

**Figure:**
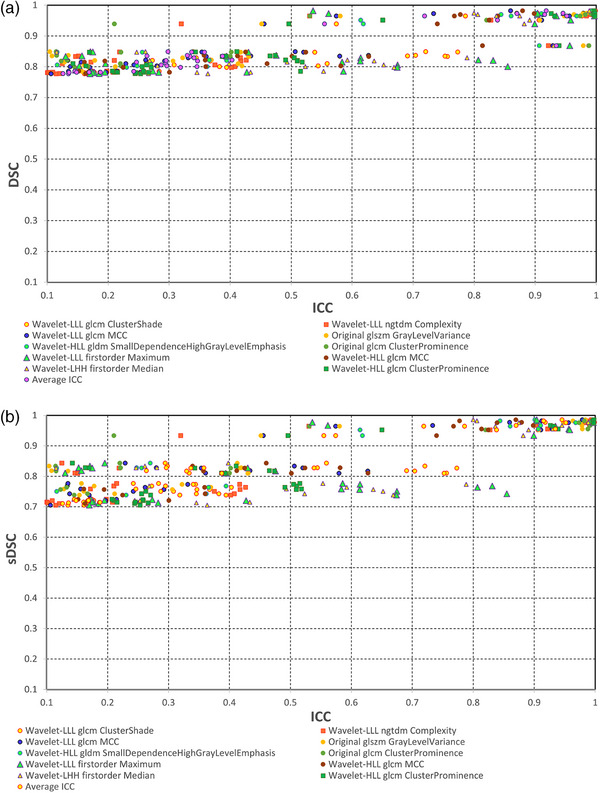

**Figure:**
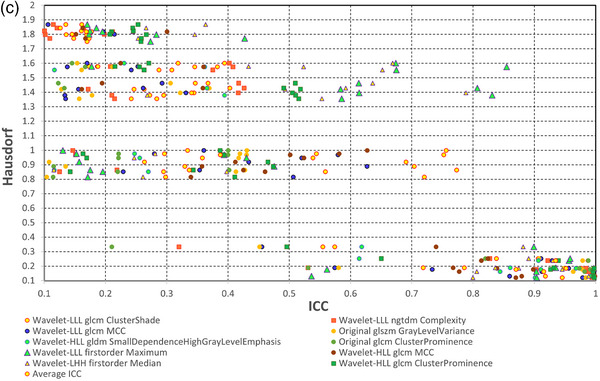

**FIGURE 5:**
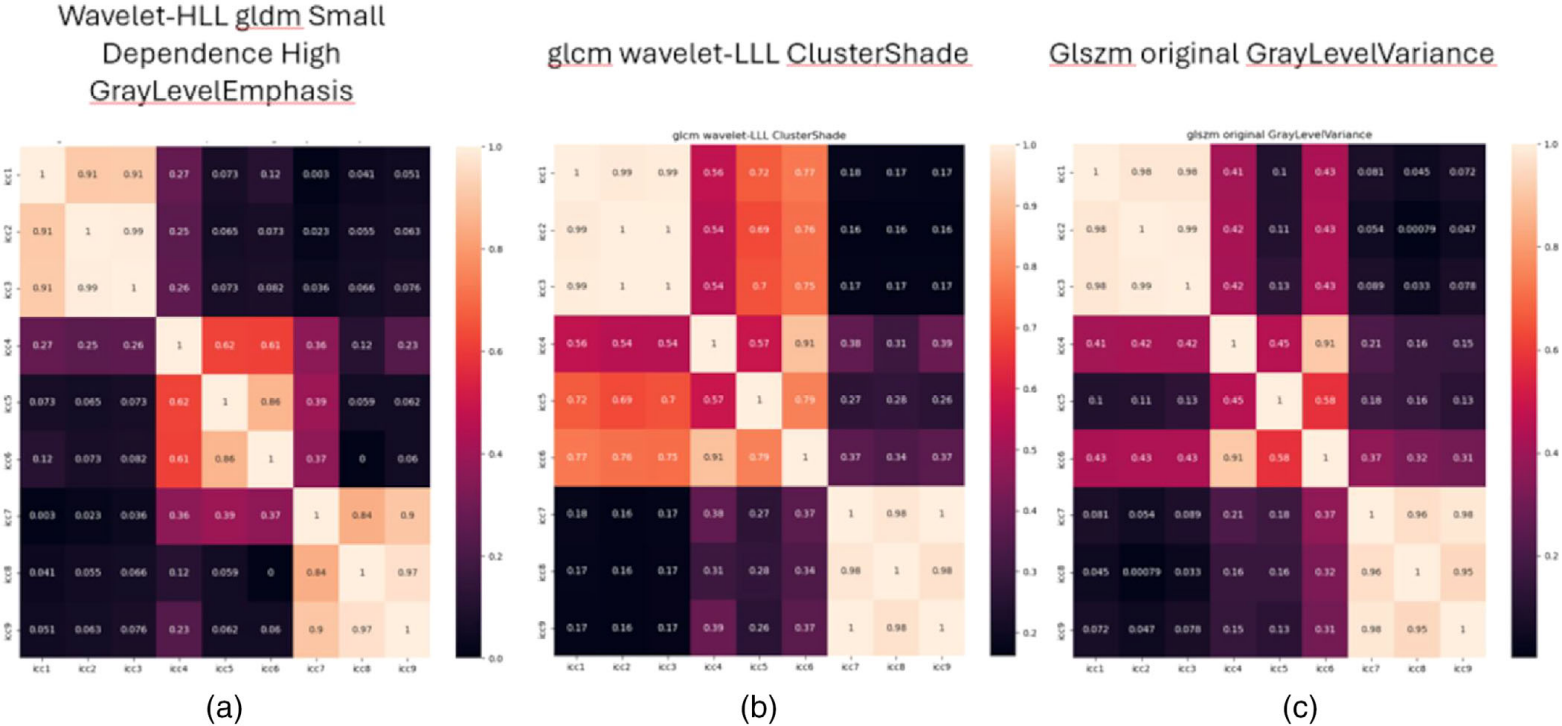
Heat maps of radiomics features: (a) Wavelet‐HLL gldm SmallDependence HighGrayLevelEmphasis, (b) Original glcm ClusterProminence, and (c) wavelet‐LLL glcm MCC.

To further demonstrate the high sensitivity of radiomics features to differences in the segmentation, we plotted heatmaps of three features (Wavelet‐HLL gldm SmallDependence HighGrayLevelEmphasis, Original glcm ClusterProminence, Wavelet‐LLL glcm MCC) in Figure 5. The figure indicated the degree of correlation between 36 pairs of 9 segmentations. The figure showed three distinguishable classes among nine segmentations. Class A: segmentations 1 to 3, Class B: 4 to 6, and Class C: 7 to 9 belonged to separate classes/groups. It is clear, especially in Figure 5a,b. The results suggest that different software and persons segmented three classes of segmentations. In other words, we can conclude that three groups created the nine segmentations because the similarity of the values for the group of the first three segmentations and the second and third groups is noticeable.

The ability of DSC, sDSC, and HD to identify the differences among segmentation methods was studied using box and whisker plots for pairs of segmentations. The results shown in Figure S1a–c in Section B of the supplemental materials indicate the limitations of these metrics compared with the radiomics features with ICC.

## 4 DISCUSSIONS

### 4.1 Example application of radiomics with ICC

In the realm of medical imaging, the assessment of tumor segmentation quality is of paramount importance. This study has convincingly demonstrated the efficacy of Radiomics features in offering a meticulous evaluation of tumor segmentations. However, it is imperative to consider alternative approaches that complement this assessment to provide a well‐rounded evaluation. The ICC is a valuable tool for measuring the agreement and consistency between different sets of segmentations. Within our context, ICC can be viewed as a benchmark for segmentation accuracy.

Imagine a scenario where ICC values between segmentation pairs, such as 1−2 or 2−3, exhibit a high level of agreement. These pairs can be deemed as reference or “Gold Standard” segmentations, signifying their accuracy and reliability in segmentation. Consequently, other segmentation methods can be evaluated by comparing their ICC values to those of the reference pairs. Segmentation techniques that closely align with these reference ICC values can be regarded as more accurate and consistent in capturing the intricacies of tumor characteristics.

For example, let us examine ICC values from the current analyses in Table 2. We arbitrarily set segmentation #1 as the gold standard. All ICC values in the columns icc1–7, 1−8, and 1−9 are less than 0.5. This implies that the segmentation method used for segmentations 7, 8, and 9 is exceptionally different from the gold standard. The segmentation method could be a physician or software. Hence, with ICC, it is easy to evaluate the performance of methods/or persons who do segmentation. If we used DSC or sDSC instead, these poor‐performing methods could be considered a good segmentation tool.

**TABLE 2:** ICC for eight pairs of segmentations of 10 selected radiomics.

Image type	Feature class	Feature name	icc1–2	icc1–3	icc1–4	icc1–5	icc1–6	icc1–7	icc1–8	icc1–9
wavelet‐LLL	glcm	ClusterShade	0.987	0.99	0.559	0.720	0.773	0.177	0.173	0.173
wavelet‐LLL	ngtdm	Complexity	0.921	0.902	0.124	0.071	0.218	0.211	0.197	0.207
wavelet‐LLL	glcm	MCC	0.929	0.733	0.228	0.506	0.353	0.196	0.214	0.252
original	glszm	GrayLevel Variance	0.978	0.978	0.412	0.104	0.427	0.080	0.045	0.071
wavelet‐HLL	gldm	SmallDependenceHighGrayLevel Emphasis	0.906	0.910	0.269	0.072	0.116	0.002	0.041	0.050
original	glcm	Cluster Prominence	0.987	0.991	0.219	0.114	0.401	0.039	0.033	0.039
wavelet‐LLL	first order	Maximum	0.956	0.561	0.195	0.170	0.174	0.194	0.173	0.177
wavelet‐HLL	glcm	MCC	0.813	0.898	0.460	0.339	0.424	0.300	0.151	0.186
wavelet‐LHH	first order	Median	0.930	0.945	0.398	0.261	0.115	0.244	0.144	0.433
wavelet‐HLL	glcm	Cluster Prominence	0.939	0.926	0.142	0.411	0.343	0.210	0.207	0.215

Abbreviation: ICC, intraclass correlation coefficient.

### 4.2 Radiomics features with ICC as similarity metrics

This study evaluated the performance of radiomics features with ICC and the most commonly applied similarity metrics, the DSC, sDSC, and HD. The results showed that DSC was greater than 0.8 for 36 pairs of segmentations, indicating good similarity. sDSC showed slightly better results, with a minimum of 0.7, yet exhibited poorer sensitivity than the radiomics features with ICC. In contrast, HD ranged from 0 to 1.9; however, the HD value is for a single point on the 3D surface of the segmentation, and the global difference cannot be properly evaluated.

It is noted that DSC, sDSC, and HD were only used to compare two segmentations of one object or tumor, for example, drawn by one master and another trainee, for their similarity. Hence, 10 of those values must be calculated for 10 tumors and two segmentations. There is no simple, readily available quantity for proper evaluation of the trainee's performance compared to the master using the segmentation data of 10 tumors. A radiomics feature value, on the other hand, is calculated for every segmentation independently, thus allowing a more statistical measure to evaluate the trainee's performance compared to the master by using a single parameter of ICC.

Furthermore, radiomics features are quantitative and high‐dimensional features extracted from medical images using advanced image processing techniques. These features capture various aspects of the tumor's characteristics, including size, shape, intensity, and texture, providing valuable information for assessing tumor properties beyond simple overlap measurements. The Radiomics features are derived from the specific segmentation area of the tumor, utilizing the region of interest defined by the physician or radiologist during segmentation. As a result, they offer a more comprehensive and detailed representation of the tumor's characteristics, contributing to a deeper understanding of the segmentation quality.

**FIGURE 6:**
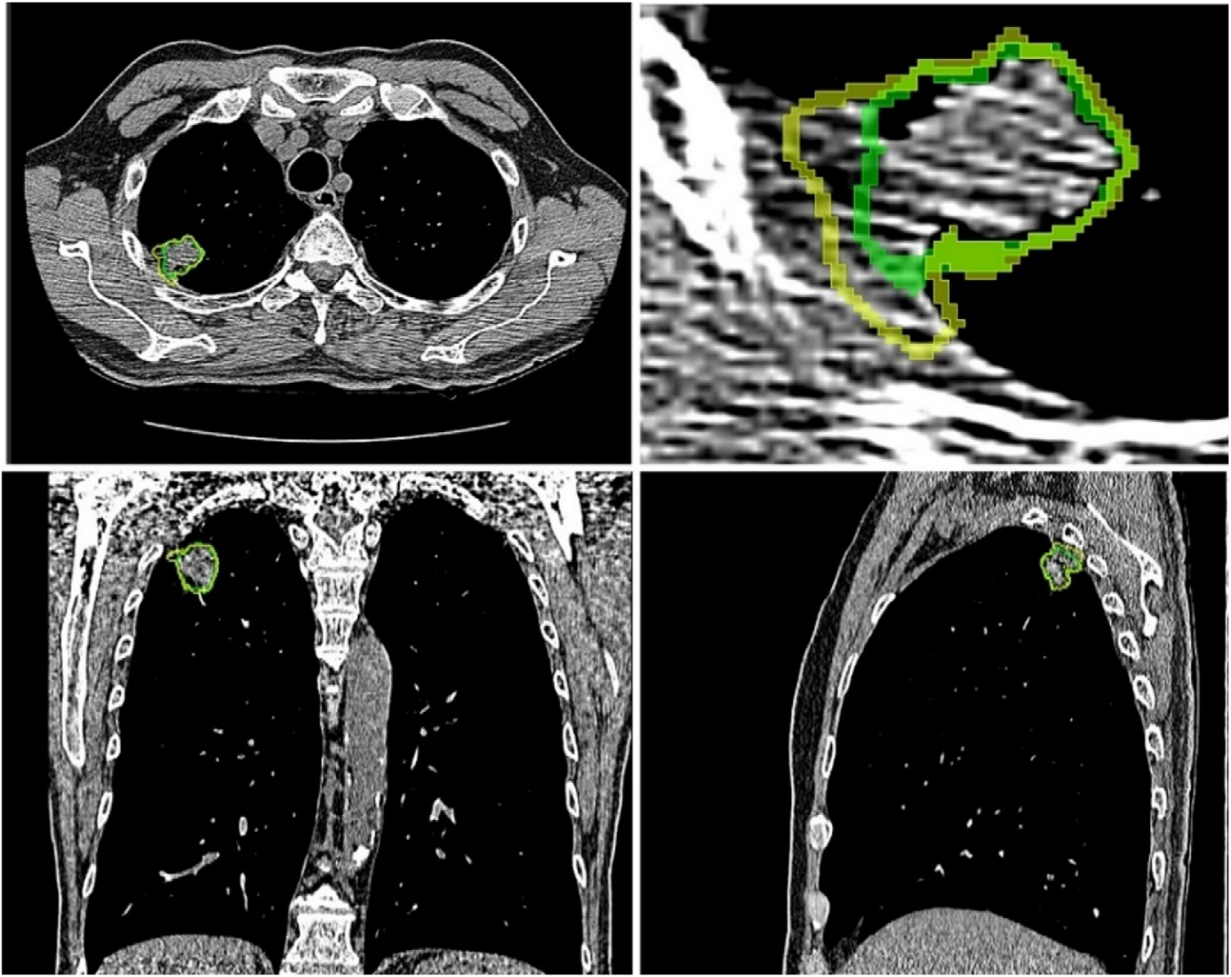
Example of two segmentations of the same tumor.

Figure 6 presents two segmentations overlayed on the same CT image data (segmentation #1 in yellow and segmentation #5 in green shaded areas). Different specialists or segmentation software created these two segmentations and did not entirely match. While the DSC indicates an 88% overlap, which is unreasonably high in this picture, the Radiomics feature's value significantly differs for these segmentations. Table 3 lists the radiomics feature values of the two segmentations for this example.

**TABLE 3:** Ten radiomics values of two segmentations for the example case.

Image type	Feature class	Feature name	Segmentation 1	Segmentation 5
wavelet‐LLL	glcm	ClusterShade	−23208.6	−26228.6
wavelet‐LLL	ngtdm	Complexity	25916.1	32854.8
wavelet‐LLL	glcm	MCC	0.6071	0.6336
wavelet‐HLL	gldm	SmallDependenceHighGray LevelEmphasis	2160.9	897.7
wavelet‐LLL	first order	Maximum	682.3	838.7
original	glcm	ClusterProminence	63701.9	71300.6
wavelet‐HLL	glcm	MCC	0.4586	0.6239
wavelet‐LHH	first order	Median	−0.0599	0.6153
wavelet‐HLL	glcm	ClusterProminence	43557.1	87644.5
original	glszm	GrayLevelVariance	74.031	76.365

### 4.3 Radiomics as an evaluation tool of segmentation ability and skills

The implications of this study extend to the realm of medical education. As budding physicians, radiologists, and healthcare professionals undergo training, acquiring accurate and precise tumor segmentation skills becomes paramount. Incorporating Radiomics features into the evaluation process, juxtaposed against the widely used DSC, can enable educators to develop comprehensive assessment tools. Medical training programs can employ this novel approach to give trainees constructive feedback on their tumor segmentation proficiency, thereby identifying areas of improvement and tailoring personalized learning plans to enhance their segmentation skills.

Beyond education, this research bears practical implications for clinical practice and quality assurance in segmentation software employing various algorithms. Healthcare facilities that utilize automated or semi‐automated segmentation software can leverage Radiomics features as an evaluation metric when comparing algorithm‐generated segmentations with human‐generated ones. This approach facilitates the identification of algorithm strengths and weaknesses, guiding further optimization and refinement. Moreover, healthcare institutions can employ this methodology to conduct routine audits, ensuring the accuracy and consistency of segmentation results across different software platforms and imaging techniques.

### 4.4 Limitations

There are several limitations to the current study. First, our application was limited to lung tumors with CT. Hence, a study is needed for tumors in different anatomical sites, and imaging techniques other than CT. Secondly, the number of patients or tumors was only 10; hence, more patient data is needed to improve the reliability of the results. Thirdly, some studies indicated the dependence of the radiomics feature values on the volume.^20^ Hence, the ICC values of radiomics features may also depend on the size of the segmented volume. The volume effects on the performance of the proposed method can only be studied with more data over a large range of segmented volumes. Lastly, the methods were applied to nine segmentations drawn by computer software. Hence, in the future, we plan to use the new evaluation tool with segmentations made by experienced radiation oncologists, radiation oncology residents, and even dosimetrists to improve their segmentation skills.

## 5 CONCLUSIONS

The findings of this study demonstrate the superiority of Radiomics features as an evaluation tool for different tumor segmentation methods in medical imaging when compared to the Dice similarity coefficient. Radiomics features provide a more sensitive and informative approach, capturing intricate details of tumor characteristics such as size, shape, texture, and intensity. Radiomics features better detect and capture subtle variations or differences in these tumor properties than the Dice similarity coefficient.

The practical application of this research includes evaluating segmentation abilities during medical training and education and identifying weaknesses in segmentation programs that employ various algorithms. Future work should explore alternative Dice similarity coefficients based on average and maximum distance, additional path length, and changes in surface, and the extension of this methodology to other anatomical locations and imaging modalities beyond lung CT imaging.

In summary, this study not only underscores the superiority of Radiomics features in segmentation evaluation but also emphasizes the significance of considering ICC indices as a complementary approach, thereby contributing to advancing tumor segmentation assessment in medical imaging.

## AUTHOR CONTRIBUTIONS

Both authors made substantial contributions to the conception and design of the work; the acquisition, analysis, and interpretation of data for the work; drafting the work and revising it critically for important intellectual content; gave final approval of the version to be published; and agrees to be accountable for all aspects of the work in ensuring that questions related to the accuracy or integrity of any part of the work are appropriately investigated and resolved.

## CONFLICT OF INTEREST STATEMENT

The authors declare no conflict of interest.

## Supporting information

Supporting Information
