# Supplementary material for: Radiomics as a measure superior to common similarity metrics for tumor segmentation performance evaluation
Source: J Appl Clin Med Phys. 2024 Jun 23;25(8):e14442. doi: 10.1002/acm2.14442 (PMC11302798; doi:10.1002/acm2.14442)

**Supplemental materials**

1. Characteristics meanings of selected radiomics features

**ClusterShade** is a feature derived from the Gray Level Co-occurrence Matrix (GLCM), which quantifies the asymmetry and the non-uniformity of the GLCM. A higher value of ClusterShade indicates more asymmetry and unevenness in the pixel intensity distribution, often pointing to more complex textures within the image.

**Cluster Prominence** is a statistical measure that reflects clusters' asymmetry and prominence within the GLCM. It quantifies the degree to which the distribution of pixel intensity values forms clusters in the matrix. A high Cluster Prominence value indicates prominent clusters within the GLCM, suggesting the presence of noticeable patterns or features within the image texture that are either very bright or dark, leading to a skewed distribution in the GLCM.

**First-order statistics** are derived directly from the pixel values of the image and do not consider the spatial relationships between pixels.

**GLDM (Gray Level Dependence Matrix)** is a texture analysis method that examines the spatial dependencies of gray levels in the image, focusing on the occurrence of specific gray level values dependent on a defined distance within the image.

**GLSZM** **(Gray Level Size Zone Matrix)** is a matrix that captures the distribution and size of contiguous regions (zones) within an image with the same gray level value.

**Gray Level Variance** is a measure derived from the GLSZM that calculates the variability of the gray level intensity within the zones. It indicates how much the gray levels differ from the average gray level within the zones. A higher variance suggests greater diversity in the gray levels across the zones, reflecting more complexity or heterogeneity in the image's texture.

**Maximum** is a first-order statistical measure that identifies the highest pixel intensity value in the image or a specific region of the image. In the context of wavelet analysis, it would be the highest pixel intensity value found in the wavelet-LLL decomposed image, representing the brightest or most intense part of that image component.

**MCC** stands for Maximal Correlation Coefficient. It is a statistical measure that quantifies the highest degree of correlation between pairs of pixel intensities in an image, indicating how predictable the relationship between different gray levels is based on the GLCM analysis. High MCC values suggest a strong correlation and structured texture in the image.

**Median** represents the middle value of the pixel intensity distribution in the decomposed image data. For the wavelet-LHH component, the median would be the middle value of the intensities in the high-frequency content, separating the higher half from the lower half of the data in terms of pixel brightness or intensity.

**NGTDM** is a texture analysis method that assesses gray level differences between neighboring pixels, and "Complexity" is a specific measure within NGTDM that quantifies the detail and intricacy of the image texture.

**Origina**l refers to the unprocessed, initial state of the image data used for analysis.

**SmallDependenceHigh GrayLevelEmphasis** feature of GLDM indicates a texture with high gray level values with small dependencies (or short distances) emphasized. It measures the prevalence of high-intensity pixels closely grouped in the image. This feature highlights areas of the image where high-intensity values occur near each other, which can be particularly prominent in the wavelet-HLL component due to its specific frequency and spatial filtering characteristics.

**Wavelet-LLL** in image analysis refers to a specific level and type of wavelet decomposition, focusing on low-frequency components in all dimensions (horizontal, vertical, and diagonal). The "LLL" indicates that low-pass filters have been applied in all directions during the wavelet transform process.

**Wavelet-LHH** indicates that the image data has been processed with a low-pass filter in one direction (usually the horizontal) and high-pass filters in the other two directions (usually vertical and diagonal). This decomposition level typically captures high-frequency content along two directions, which can represent edges or fine details in those orientations.

**Wavelet-HLL** refers to a wavelet transform level where 'H' stands for high-pass filtering, and 'LL' indicates low-pass filtering applied in different directions. This level captures details that are high in one dimension and smooth in the others.

1. Supplemental figures

Figure S1. Boxplot comparison for pairs of contours 1 and 2, 1 and 4, and 1 and 7 for (a) Dice Similarity coefficient, (b) Surface Dice coefficient, and (c) Hausdorff coefficient

1.
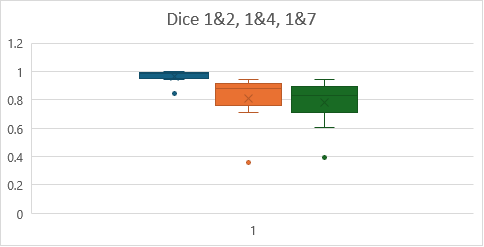

2.
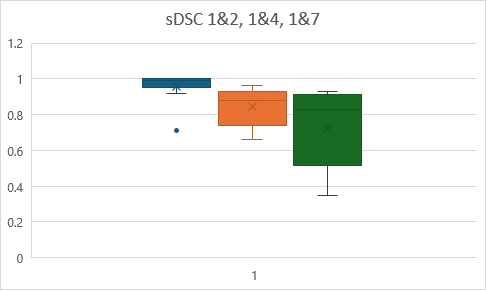

3.
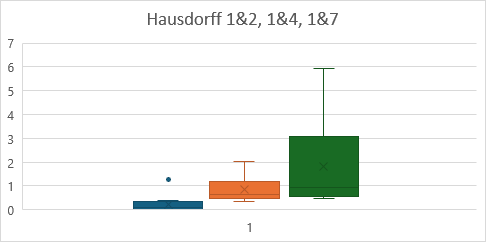

Supplement: Supplementary file 1 — Supporting Information [file ACM2-25-e14442-s001.docx]
